# Global environmental equities and investor sentiment: the role of social media and Covid-19 pandemic crisis

**DOI:** 10.1007/s11846-022-00614-9

**Published:** 2023-01-03

**Authors:** Vítor Manuel de Sousa-Gabriel, María Belén Lozano-García, Maria Fernanda Ludovina Inácio Matias, Maria Elisabete Neves, Jennifer Martínez-Ferrero

**Affiliations:** 1 Centro de Estudos e Formação Avançada em Gestão e Economia (CEFAGE), Escola Superior de Tecnologia e Gestão, Instituto Politécnico da Guarda, Guarda, Portugal; 2 Instituto Multidisciplinar de Empresa, Faculdad de Economia e Empresa, Universidade de Salamanca, Salamanca, España; 3 Escola Superior de Gestão, Hotelaria e Turismo, Universidade do Algarve, Faro, Portugal; 4 Polytechnic of Coimbra, Coimbra Business School Research Centre, Centre for Transdisciplinary Development Studies, Coimbra Portugal and UTAD|CETRAD, Vila Real, Portugal

**Keywords:** Environmental investment, Investor sentiment, Social media, Pandemic crisis, 62, 91

## Abstract

According to researchers, information generated from social media provides useful data for understanding the behaviour of various types of financial assets, using the sentiment expressed by these network users as an explanatory variable of asset prices. In a context in which investment based on sustainability and environmental preservation values is vital, there is no known scientific work that analyses the relationship between social networks and environmental investment, which is closely related to the 2030 Agenda for Sustainable Development. In this study, we aim to identify how investor sentiment, generated from social networks, influences environmental investment and whether this influence depends on the time variable, as well the role of the pandemic crisis and the Russia-Ukraine war. Our results show different forms of behaviour for the different periods considered, with the proximity between the two types of variables being time-varying. For shorter periods, proximity occurred mainly during the pandemic crisis, repeatedly revealing that sentiment is a risk factor in environmental investment and in particular how important the information generated from social networks can be in pricing environmental assets. For longer periods, no common stochastic trends were identified. The mechanisms generating the series are thus characterised by a certain autonomy.

## Introduction

Market efficiency, investor rationality, and asset price capacity to reflect the most relevant market information form the main premises of classical financial theory. In an efficient market, asset prices are defined by the current value of future cash flows, which is why asset prices are not felt to be affected by investor sentiment. However, the history of financial markets reports numerous cases where the behaviour of asset prices does not always find a satisfactory explanation in classical theory, above all in periods of speculation and crisis.

Kahneman and Tversky (1974), Shiller (1981, 2006), Mandelbrot (2006), and Nofsinger (2014), among other authors, have contributed to developing the field of behavioural finance, which has questioned the efficient market hypothesis, rejecting the assumption of perfect investor rationality, since the latter’s decisions are influenced by their own feelings, which results in a certain degree of irrationality in the decision-making process.

Society’s perception of company responsibility has evolved significantly over time. Neoclassical theory, as proposed by Friedman (1970), argues that companies’ sole responsibility is to maximize shareholder value and that social responsibility represents an additional cost for firms, penalizing their objectives to maximize profit. According to this perspective, social responsibility practices restrict companies’ possibilities for economic growth, particularly when compared to other competing firms that do not value the same type of practices. Contrary to neoclassical theory, stakeholder theory, whose main proponent is Freeman (2008), supports the thesis that companies should not only answer to their shareholders but also to other stakeholders, such as creditors, collaborators, suppliers, customers, and society as a whole, in an attempt to satisfy all interests.

The combination of various urgent issues, such as the shortage of water, environmental disasters, global warming, human rights, and poverty, as well as the occurrence of scandals related to bad governance or even episodes of the financial crisis, have all contributed to stakeholders attaching greater value to the question of sustainability since this also adds value to the investment in the long term (Porter and Kramer 2006; KPMG 2011).

When adapting to changes in management models, financial markets have followed an evolutionary process. That evolution is seen in the emergence of investment alternatives; namely, those involving so-called socially responsible investment, which merges financial objectives with environmental, social, and governance objectives (Statman and Glushkov 2008; Renneboog et al. 2008; Lombardo and D'Orio 2012; Ng and Zheng 2018). This type of investment has gained relevance in the global context and is gradually arousing investor interest almost everywhere (GSIA 2019). For example, Findlay and Moran (2019) argue that impact investing is under-institutionalized and is in a legitimacy-building phase. Also, Gao, Li, and Wang (2021), in a recent paper, explored the interaction between investor attention and green security markets, including green bonds and stocks.

The effect of investor sentiment on stock markets has led to several studies. However, the great majority have focused mainly on the US market and traditional stock indices, based on a purely financial logic rather than on other markets and investment segments (Baker et al. 2012; Bathia and Bredin 2012). Little research has been devoted to sustainable investment, with most of it focusing above all on its performance concerning traditional indices. It is, nevertheless, important to understand the behaviour mechanisms of sustainable indices and their various segments. The aim here is to fill an empirical gap in research, focusing on the relationship between investor sentiment and a segment of sustainable investment, adopting both a short-term and long-term perspective. To do so, the environmental investment segment is analysed by selecting five global indices; namely, alternative energy, sustainable water, green construction, clean technology, and pollution prevention, as well as an indicator of investment sentiment, covering a period of approximately twelve years from January 2009 to March 2022. This helps us to obtain indications about what impact investor sentiment has on environmental investment.

As regards the methodology, and as a core element of the study, we use the Twitter happiness index as a proxy of investors’ online sentiment, in line with the work by Zhang et al. (2016), Shen et al. (2018) and Teti et al. (2019), Shiva, and Singh (2020), and Chen, Lai, and Cai (2021), which can be considered as an indicator of global sentiment, and taking into account the role of social media as a tool for investing and predicting (Oh and Sheng 2011; Piñeiro-Chousa et al. 2017). In addition, as it is a daily index, it will help to capture emotions at each moment more suitably, as opposed to most sentiment indicators considered in other studies such as those by Lemmon and Portniaguina (2006), Schmeling (2009) and Bathia and Bredin (2012), who use information with a monthly frequency but which is not able to reveal the momentum effect in the short term.

In order to examine the short and long-term dynamics between sentiment and investment, we apply various statistical tools. To analyse short-term dynamics, we use some of the most recent bivariate models of conditional heteroscedasticity (GARCH-DCC and GARCH-cDCC), which give a more detailed perception of the dynamics generated between the two variables studied, improving performance estimates, above all compared to models assuming a constant conditional correlation. Concerning the possible existence of long-term balances, we apply the methodological proposal of Pesaran et al. (2001), in the form of ARDL (autoregressive distributed lag) and Bounds tests.

In sum, this research aims to expand the existing financial literature, empirically and methodologically. We believe it is important to go deeper into the factors that influence the behaviour of asset prices, as this is a crucial element in the sphere of finance (McGurk et al. 2020). Despite being relatively understudied and breaking with traditional theory, investor sentiment is one factor that is of special interest to academics, investors, and regulators alike. In addition, the market indices generally used in the literature to explore these factors are the so-called traditional indices, created in line with purely financial assumptions. Therefore, this study presents two combinable and differentiating elements which have not yet been exhaustively studied in the literature, i.e., including investor sentiment in determining asset prices, and analysing its relationship with environmental investment, which complies with the Sustainable Development Goals (SDGs) of the United Nations 2030 agenda. Specifically, we conjecture as to whether the time factor—short-term and long-term—influences the relation between environmental investment and investor sentiment, considering the assumptions of contemporaneity and lagging in the relations created between the two variables in the period spanning January 2009 to March 2022, with special emphasis on the pandemic crisis and the recent war conflict between Russia and Ukraine.

In terms of structure, this research continues in Sect. 2 with a literature review. Section 3 describes the data and the methodology. Section 4 presents the empirical results and findings obtained and provides a discussion. Finally, Sect. 5 presents the final considerations.

## Literature review

### Investor sentiment and stock markets

In the second half of the last century, the financial theory was based on two central approaches: classical and behavioural. The former was dominant in the 1960s, while the latter emerged in the 1980s (Shiller 2006).

The classical approach assumes investor rationality, such that their decisions are explained by the risk-profitability binomial (French et al. 1987; Statman 2005). It is also assumed that the investor will try to maximize the available arbitrage opportunities and that asset prices converge towards their fundamental value as a consequence of competitive interaction among investors (Shleifer and Vishny 1997; Baker and Wurgler 2007). In various studies, the assumptions defined by the classical approach have been questioned due to the recognition of market anomalies, particularly during phases characterized by high volatility in asset prices (De Bondt et al. 2008; Statman 2005, 2008). In an effort to remedy the supposed limitations of the classical approach, the behavioural approach, also known as behavioural finance (Barberis and Thaler 2003), was developed to explore what effect issues of a psychological nature might have on investors’ decision-making processes and the behaviour of financial markets (De Bondt et al. 2008; Shiller 2006). The behavioural approach is based on two central assumptions. The first is the existence of limits to investors’ rationality due to cognitive bias caused by the effect of investors’ emotions on their decision-making, with implications for the behaviour of asset prices which can generate waves of sentiment and market bubbles (Statman 2005; De Bondt et al. 2008; Schmeling 2009; Finter et al. 2011). According to Zouaoui et al. (2010), these waves of sentiment and irrationality are the consequence of over-optimistic or over-pessimistic expectations to the point that they affect asset prices for long periods and in some cases trigger crises. The second assumption is associated with the limits to arbitrage arising from the existence of costs and restrictions associated with market transactions (Barberis and Thaler 2003).

The literature on behavioural finance has paid special attention to the relationship formed between stock market profitability and investor sentiment. This is understood as a generalized mindset among investors regarding cash flows and future investment risks and which lacks any justification in the fundamental information known (Baker and Wurgler 2007), above all when seeking to understand to what extent the second variable helps explain the behavior of the first (Brown and Cliff 2004; Schmeling 2006; Finter et al. 2011; Baker et al. 2012; Bathia and Bredin 2012; Oliveira et al. 2013; Fang et al. (2018); Escobari and Jafarinejad 2019).

Results obtained from the previously mentioned works on the explanatory power of the sentiment variable in stock markets do not concur, probably due to the use of different sentiment measures, sample periods, market segments, and methodological procedures. For example, Brown and Cliff (2004) and Oliveira et al. (2013) conclude that, in the short run, the explanatory power of the sentiment variable on stock market returns is little or null.

Schmeling (2009), Baker et al. (2012), Bathia and Bredin (2012) and Herve et al*.* (2019), among other authors, concluded that investor sentiment can help predict stock market profitability and that the relationship formed between the variables is negative. In the context of behavioral finances, this situation is explained by the presence of less-informed investors, so-called noise traders, who do not base the respective investment-decision processes on fundamental information but are guided by emotions. This situation is caused by persistent waves of sentiment that make asset prices deviate from their fundamental value (Schmeling 2009; Finter et al. 2011). Noise-trader participation in the market creates noise in asset prices, not only contributing to increased profitability of informed transactions but also to their risk (Black 1986; De Long et al. 1990; Peress and Schmidt 2017; Lin, Sias, and Wei 2018). According to Schmeling (2009), asset price deviations from their respective fundamental value follow a path of adjustment, where high (low) levels of investor optimism lead to low (high) profitability. In the case of a significant price deviation from the respective fundamental value, investors believe that the risk of adopting a position against noise traders is justified because of high future profitability, which leads them to become more aggressive when such deviations are recorded (Baker et al. 2012).

### Measuring investor sentiment

Studying the impact of investor sentiment on market behaviour begins by measuring this variable. In this regard, several measures or proxies are available to meet this objective. For example, Aggarwal (2019) develops a literature review study on different sentiment measures in financial literature. Nevertheless, these measures face several obstacles. First, they do not allow sentiment to be measured with a high level of accuracy (Baker et al. 2012; Finter et al. 2011), and second, there is no universally accepted measure from which evaluations and comparisons can be made (Bathia and Bredin 2012; Corredor et al. 2013).

Studies carried out in this field have used two major groups of measures: direct and indirect. The former seeks to measure investor sentiment through the use of questionnaires. These surveys are based on investors being asked about their degree of optimism or pessimism regarding economic and market conditions, which allows us to examine how investors make their financial decisions (Baker and Wurgler 2007). The latter seeks to measure investor sentiment from market variables in order to infer generic investor sentiment.

The technological revolution that has occurred in recent decades, namely the constant interaction with technological systems that implies the widespread presence of computers and the internet, has given rise to a wealth of data that can be used to analyse the collective behaviour related to a wide range of topics (Vespignani 2009; King 2011). Recently, the repository of internet activity has been used to understand the concerns, perceptions, and interests of the global population, serving as a basis to study various areas of knowledge, particularly social, economic, and financial sciences. Over the last decade, several researchers have proved that investor sentiment, obtained from information generated on blogs and social media, has the ability to help predict stock market behaviour (Oh and Sheng 2011; Zhang et al. 2011; Bollen et al. 2011; Piñeiro-Chousa et al. 2017). For example, Piñeiro-Chousa et al. (2021) argue that stock market price prediction has improved due to social network activity as these networks provide useful information such as message sentiment.

Several scientific papers have studied the relationship between internet data and financial markets, considering several types of data: internet news (Alanyali et al. 2013; Lillo et al. 2015), search engine queries (Curme et al. 2014; Varkman and Kristoufek 2015), and social media. As regards the data generated from social media, the Twitter platform has been used in recent years to help make financial forecasts (Ranco et al. 2015; Zhang et al. 2016; Shen et al. 2018; Teti et al. 2019). Zhang et al. (2016) analyzed relationships between daily Twitter sentiment and the performance of eleven international stock markets using Granger causality and cross-sectional analysis and concluded that there is a significant dependence between online sentiment and stock market performance in general. Shen et al. (2018) resorted to daily Twitter sentiment as a proxy for online sentiment dynamics to investigate its link to the skewness of the profitability of several international stock markets, dividing daily sentiment into quantiles from the least happy days to the happiest. The conclusion was that skewness around the happiest days is significantly higher than the skewness around the least happy days. In turn, Teti et al. (2019) started using Twitter as a source of sentiment data to explore its ability to predict the performance of the North American tech stock sector, applying OLS models. These authors concluded that there is a statistically significant relationship between the variables studied.

Following the above, and considering that investor sentiment cannot be directly considered as measurable or observable, our proxy for investor sentiment corresponds to the daily happiness index derived from the website https://hedonometer.org/api.html. The raw daily happiness scores are extracted by means of a natural language processing technique based on a random sampling of about 10% (50 million) of all messages posted in Twitter’s Gardenhose feed. In order to quantify the happiness of the atoms of language, Hedonometer.org merged the 5000 most frequent words from a collection of four corpora: Google Books, New York Times articles, Music Lyrics, and Twitter messages. The result is a composite collection of approximately 10,000 unique words. Then, using Amazon’s Mechanical Turk service, Hedonometer.org had each of these words scored on a nine-point scale of happiness, with 1 corresponding to “sad” and 9 to “happy”. Words in messages written in English (containing about 100 million words per day) are assigned a happiness score based on the average happiness score of the words contained in the messages.

### Sustainable investment

Concerns about global warming and climate change, the environment and water scarcity, human rights, scandals, and financial crises, among other factors, have made sustainability a central issue today. The growing global awareness of sustainability issues has been particularly marked by certain initiatives that have had a catalytic effect on public opinion and decision-makers. Among the various initiatives implemented in recent years, of particular note is the Agenda 2030, launched in September 2015 by the United Nations, which defined the 17 Sustainable Development Goals (SDGs), emphasizing the importance of raising awareness in the financial system and socially responsible investment to meet these supranational ambitions. Socially responsible investment, also called sustainable investment or ethical investment, has encouraged academics, politicians, and researchers to find a more sustainable path for the planet and economies. The attention given to the subject of sustainability has led to the emergence of various sustainable stock market indices and has increased the weight of this investment segment in the global context.

The subject of sustainable stock market investment is relatively recent, with the first sustainable index appearing in 1990, called the Domini 400 Social Index (DSI). Over the last fifteen years, various indices have been created in this area, prominent amongst which are the Dow Jones Sustainability Index (DJSI), FTSE4Good Index, E. Capital, Ethibel, Humanix, Jantzi, KLD Analytics and Morgan Stanley Capital International (MSCI). As for strengthening the weight of sustainable investment, it should be highlighted that the value of global assets in this investment sector has grown significantly in recent years, from a total of 13.3 trillion dollars in 2012 to 30.7 trillion dollars in 2018.

Although sustainable investment has aroused great interest among investors and academics, little scientific research has been devoted to the subject. The vast majority of studies on sustainable investment have focused on its performance compared to traditional investment (Bauer et al. 2007; Skare and Golja 2012; Martinez-Ferrero and Frias-Aceituno 2015; Marti et al. 2015; Ransariya and Bhayani 2015), with a limited amount of scientific research addressing the behavior of sustainable indices. Central among these studies are Roca et al. (2010), Gabriel and Pazos (2017, 2018). The former analyzed the short-term links between various sustainable indices and concluded that these have intensified over time. The latter analysed the dynamics created in the short and long term between environmental segments and concluded that these segments display very similar behaviour in the short term, which does not allow any differentiation from traditional indices to be inferred. In the long term, no balanced relations were identified.

As far as we know, there are no studies that consider and analyse the relationship between investor sentiment -through an analysis of social networks-, and sustainable investment -a topic that is especially relevant in the current context of Sustainable Development Goals-. Nevertheless, and after considering the work presented above, we can conclude that although some studies have examined investor sentiment with stock market performance additional research is still needed. A more in-depth analysis of environmental indices and the time-varying relationship between investor sentiment and the stock market has not been considered in previous studies.

For this reason, we chose to study the aforementioned relationship between two types of variables. This allows us to analyse investor behaviour using a recent variable of social activity, which is highly reliable for measuring a new dimension of financial markets, and it also enables us to monitor the trend and evolution of sustainable investment.

Based on the literature review and considering the research objectives, we formulated five research questions as a means to gain insight into the behavioral mechanisms of environmental investment, which is aligned with the sustainability objectives advocated in the 2030 agenda:

i. Investor sentiment is linked to environmental investment?
ii. The links between investor sentiment and environmental investment remain stable over time?
iii. Does investor sentiment affect all environmental investment segments in the same way?
iv. Is investor sentiment immediately incorporated into the share prices of environmental investment segments?
v. Has the pandemic crisis changed the links among sentiment and environmental investment segments?

## Data and methods

### Data

To fulfil the aims of the research, the methodology chosen is applied to daily frequency data for a period of approximately twelve years from January 2009 to March 2022 involving five global environmental indices that are in line with the United Nations' SDGs and a series regarding investor sentiment. Table 1 provides a brief description of each index.

**Table 1:** Indices and variable descriptions

Index	Description	
Alternative Energy	AE	Includes developed and emerging market large, mid and small-cap companies that derive 50% or more of their revenues from products and services in Alternative energy (Goal 7). It was launched on 20 January 2009. On 28 March 2022, it was composed of 80 constituents from the utilities, industrials, information technology, energy and materials sectors
Energy Efficiency	EE	Includes developed and emerging market large, mid and small-cap companies that derive 50% or more of their revenues from products and services in Energy Efficiency (Goal 9). It was launched on 20 January 2009. On 28 March 2022, it was composed of 59 constituents from the consumer discretionary, industrials, information technology, real estate, materials and utilities sectors
Green Building	GB	Includes developed and emerging market large, mid and small-cap companies that derive 50% or more of their revenues from products and services in Green Building (Goal 11). It was launched on 20 January 2009. On 28 March 2022, it was composed of 83 constituents from the real estate and consumer discretionary sectors
Pollution Prevention	PP	Includes developed and emerging market large, mid and small-cap companies that derive 50% or more of their revenues from products and services in Pollution Prevention (Goal 12). It was launched on 20 January 2009. On 28 March 2022, it was composed of 6 constituents from the materials, consumer staples and industrial sectors
Sustainable Water	SW	Includes developed and emerging market large, mid and small-cap companies that derive 50% or more of their revenues from products and services in Sustainable Water (Goal 6). It was launched on 20 January 2009. On 28 March 2022, it was composed of 9 constituents from the utilities, industrials, information technology and materials sectors
Happiness Sentiment	HS	Is generated from the Twitter Gardenhose feed database, a randomly selected database with 50 million (10% of the total) Twitter posts. It was launched on 9 September 2008. With this enormous volume of Twitter posts, index makers use approximately 10,000 sentiment-related words, such as love, happy, among others, along with the natural language process techniques provided by Amazon’s Mechanical Turk service, for the quantification of the happiness score of Twitter posts

The series relating to the two variables considered in this work were obtained from MSCI (environmental indices) and the Twitter Gardenhose feed database (happiness sentiment).

To analyse the main statistical properties of the two series, the logarithmic variation was considered. The daily values of the environmental indices and economic sentiment were transformed into variation series, $r_{t}$, by applying the expression $\ln \left( {{{P_{t} } \mathord{\left/ {\vphantom {{P_{t} } {P_{t - 1} }}} \right. \kern-0pt} {P_{t - 1} }}} \right)$, where $P_{t}$ and $P_{t - 1}$ represent the daily values of a given series on days $t$ and $t - 1$, respectively.

### Methods

The Bounds Test was used to analyse whether the time factor – long-term and short-term – influences the relation between environmental investment and investor sentiment, following the method proposed by Pesaran et al. (2001), as well as two multivariate models of conditional heteroscedasticity.

#### Multivariate models of dynamic conditional correlation

In order to examine the dynamic contemporary and lagged links between investment and investor sentiment, we use two multivariate models of conditional heteroscedasticity; specifically the variants GARCH-DCC and GARCH-DCC-DECO. The aim is to ensure parsimony in the estimations without neglecting the indications of the usual information criteria of Akaike and Schwarz.

As do Dajcman et al. (2012), Lee and Jeong (2014), Cai et al. (2016), and Gabriel and Pazos (2017, 2018), we selected conditioned heteroscedasticity multivariate models to adequately accommodate the time-varying volatility characterizing the financial series and to analyse any time-varying correlations established between investor sentiment and environmental indices.

Engle (2002) proposed a multivariate model of dynamic conditional correlation (GARCH-DCC), which differs from other models, for example, the constant conditional correlation proposed by Bollerslev (1990), by allowing the conditional correlation matrix to be variable over time.

This model is estimated in two stages. In the first stage, univariate GARCH models are applied to each series. In the second, the standardized residuals obtained in the first stage are used to obtain the conditional correlation.

In the GARCH-DCC model, the variance–covariance matrix is written as:

1 $$H_{t} = D_{t} R_{t} D_{t}$$

$D_{t}$ is the diagonal matrix of the time-varying standard deviations from the univariate GARCH estimations and $R_{t}$ is the matrix of correlations variable over time. $R_{t}$ can be defined as:

2 $$R_{t} = {\text{Q}}_{t}^{* - 1} {\text{Q}}_{t} {\text{Q}}_{t}^{* - 1}$$

3 $$Q_{t} = \left( {1 - \theta_{1} - \theta_{2} } \right)Q_{t}^{*} + Q_{t} Q_{t}^{* - 1} \theta_{1} \varepsilon_{t - 1} \varepsilon^{^{\prime}}_{t - 1} + \theta_{2} Q_{t - 1}$$

$Q_{t}$ is the unconditional variance between the series and ${\text{Q}}_{t}^{*}$ is the unconditional covariance between the series, and $\varepsilon_{t - 1}$ is the empirical matrix of standardised residuals. $\theta_{1}$ and $\theta_{2}$ are implying the persistence of shocks. The sum of them, measures volatility persistence. It is expected that $\theta_{1} \ge 0$, $\theta_{2} \ge 0$ and $\theta_{1} + \theta_{2} < 1$ for the conditional correlation matrix to be defined as positive.

However, there are some issues about the consistency of the DCC-GARCH model. Aielli (2013) reports that an estimation of the empirical correlation matrix is inconsistent because:

4 $$E\left[ {\varepsilon_{t} \varepsilon_{t}^{^{\prime}} } \right] = E\left[ {E_{t - 1} \varepsilon_{t} \varepsilon_{t}^{^{\prime}} } \right] = E\left[ {R_{t} } \right] \ne E\left[ {Q_{t} } \right]$$

Aielli (2013) suggest the consistent estimator of dynamic correlations that named as corrected DCC.

(cDCC) to overcome this issue. The cDCC model improved consistency by reformulating the correlations as:

5 $$Q_{t} = \left( {1 - \theta_{1} - \theta_{2} } \right){\text{Q}}_{t}^{*} { + }\theta_{1} \left\{ {{\text{Q}}_{t - 1}^{{{1 \mathord{\left/ {\vphantom {1 2}} \right. \kern-0pt} 2}}} \varepsilon_{t - 1} \varepsilon_{t - 1} {\text{Q}}_{t - 1}^{{{1 \mathord{\left/ {\vphantom {1 2}} \right. \kern-0pt} 2}}} } \right\} + \theta_{2} Q_{t - 1}$$

Equation (5) shows that in the cDCC model conditional correlations formulated with the combination of the relevant innovations and past correlations. When the persistence of the correlation and effects of the innovations are sufficiently high, the cDCC estimator is still unbiased, but DCC estimator is not.

#### Cointegration tests

The Johansen (1988) test, possibly the most commonly used in analysing cointegration, allows the long-term balanced relations between different variables to be studied, requiring them to be integrated at least in first differences I (1). However, if the variables present different levels of integration, such as I(0) and I(1), the usual cointegration tests produce biased results. To overcome this limitation, Pesaran et al. (2001) developed the ARDL test, also known as the Bounds Test. One of the main advantages of this test is that it avoids the problem of endogeneity since all the variables are assumed to be endogenous. There is also the possibility of variables of order one and zero co-existing in the model, which circumvents the structural limitations inherent to other approaches. However, the Bounds Test does not function correctly when variables of order above one are included, which is its main limitation.

In a bivariate case, the ARDL model with an error-correcting mechanism (ECM) can be expressed in the following equation:

6 $$\Delta \ln Y_{t} = \alpha_{0} + \sum\limits_{i = 1}^{p} {\delta_{1} } \Delta X_{t - i} + \sum\limits_{i = 1}^{q} {\delta_{2} } \Delta Y_{t - i} + \alpha_{1} \ln Y_{t - 1} + \alpha_{2} \ln X_{t - 1} + \varepsilon_{t}$$

where, $\Delta$ is the operator of the first difference, $Y_{t}$ is the dependent variable, $X_{t}$ is the independent variable, $\alpha_{0}$ is the constant of the model, $\delta_{1}$ and $\delta_{2}$ are the short-term dynamic coefficients, $\alpha_{1}$ and $\alpha_{2}$ long-term multipliers, and $\varepsilon$ is random disturbance.

The null hypothesis of non-cointegration between the variables studied is given by $H_{0} :\alpha_{1} = \alpha_{2} = 0$, as opposed to the alternative hypothesis, $H_{a} :\alpha_{1} \ne \alpha_{2} \ne 0$ of the variables not being cointegrated.

## Empirical results and discussion

**Fig. 1:**
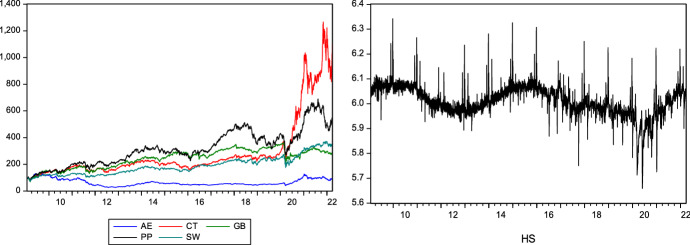
Evolution of stock market indices and happiness sentiment

The evolution of the six series in levels (environmental indices, on the left; happiness sentiment, on the right), in the period between January 2009 and March 2022, is shown in Fig. 1.

Graphical analysis of stock markets leads to the conclusion that over the sample period these present fairly similar patterns of behaviour, except for the alternative energy index. Visual analysis from Fig. 1 helps to form an initial perception of the evolution over time of the variables studied but does not allow any inference of the relations formed between them. In the next section of the study, we conduct a more in-depth analysis of the co-movements generated between the two variables in the short and long term.

The main descriptive statistics of the logarithmic variations of the stock market indices and investor sentiment are presented in Table 1. Analysis of these statistics leads to the initial conclusion that, except for the investor sentiment indicator and the alternative energy segment, the series presents positive average daily profitability. We also observe that the clean technology segment, related to Industry, Innovation, and Infrastructure (Goal 9), shows the largest mean returns, while the alternative energy segment, related to Affordable and Clean Energy (Goal 7), presents the lowest return. However, based on the analysis of the variance test, the null hypothesis proposing that the series have the same mean is not rejected. As regards the standard deviation and taking into account the Levene test, the null hypothesis of equality of variances is rejected, which leads us to conclude that there are statistically significant differences.

The Jarque–Bera adherence test was used to understand the suitability of the adjustment of the normal distribution to the empirical distributions of the profitability series. The statistical probabilities are presented in Table 1. The results obtained allow us to conclude that these series mostly display statistical significance at 1%, such that the hypothesis of normality is rejected.

The Ljung–Box test for up to 20 lags indicates the presence of significant linear and nonlinear dependencies in the returns of all the series. In turn, the ARCH test reveals the presence of heteroscedasticity in all series. These results suggest the need to study further the dynamics generated between the two types of variables considered in this research.

To study the stationarity of the series at levels and the profitability series, the traditional Augmented Dickey-Fuller tests (ADF) were applied. The null hypothesis $\left( {H_{0} } \right)$ of this test stipulates that the series has a unit root, i.e., that the series is integrated of order 1, I (1), compared to the alternative hypothesis $\left( {H_{a} } \right)$ of the series not having a unit root or being I(0). The results of the series stationarity tests are presented in Table 2. The series in levels of the stock market segments and investor sentiment are found to be non-stationary, i.e., I(1), for a 1% level of significance, while the series of logarithmic variations show stationarity, I(0), for the same level of significance.

**Table 2:** Descriptive profitability statistics

	AE	CT	GB	PP	SW	HS	Equality Test (Prob.)
Mean	−0.00001	0.00071	0.00033	0.00051	0.00038	−0.00001	(0.14143)
Standard deviation	0.01531	0.01542	0.01267	0.01401	0.01257	0.00541	(0.00000)*
Asymmetry	−0.32584	−0.12654	−0.75187	−0.59808	−0.47012	−0.97385	
Kurtosis	7.46208	10.67028	16.90202	9.54682	12.12666	13.92794	
JB	(0.00000)	(0.00000)	(0.00000)	(0.00000)	(0.00000)	(0.00000)	
LB (20)	(0.00000)*	(0.03102)**	(0.00000)*	(0.00000)*	(0.02391)**	(0.00000)*	
LB^2^ (20)	(0.00000)*	(0.00000)*	(0.00000)*	(0.00000)*	(0.01623)**	(0.00000)*	
ARCH (1)	(0.00000)*	(0.00000)*	(0.00000)*	(0.00000)*	(0.00000)*	(0.00000)*	
ADF (Levels)	(0.58220)	(0.04135)**	(0.12834)	(0.72745)	(0.00674)	(0.22934)	
ADF (Prof.)	(0.00000)*	(0.00000)*	(0.00000)*	(0.00000)*	(0.00000)*	(0.00000)*	

This table contains the descriptive statistics for the daily returns series for the Alternative Energy Indices (AE), Clean Technology (CT), Green Building (GB), Pollution Prevention (PP), Sustainable Water (SW), and the Happiness Sentiment (HS), for the sample period from January 2009 to March 2022. The last column reports the mean and variance equality tests for the environmental indices, using the ANOVA and Levene statistics, respectively. LB (20) and LB^2^ (20) are Ljung–Box tests for 20th‐order serial correlation in the returns and squared returns. ARCH (1) is the Engle test for the first‐order ARCH. The ADF statistic tests the stationarity of the series. Values in parentheses indicate the p-value. *, and ** indicate significance at the 1% and 5% levels, respectively

To further the study of the short-term dynamics between environmental investment and investor sentiment, various bivariate models of conditional heteroscedasticity were estimated, starting from the series generated for the logarithmic variations, after confirming their stationarity, and considering the results of the ADF tests reported above in Table 2.

Estimations of the bivariate models involved various specifications (GARCH-DCC and GARCH-cDCC); namely, including the asymmetric effect, the size of the lag, and the statistical distribution of errors, and always seeking to respect the specific assumptions of the models in question. However, it is important to mention that only the inclusion of the asymmetric effect helped, in some cases, to improve the estimating performance of the models, such that in general the respective simpler versions were estimated. Considering the information criteria of Schwarz and Akaike, the GARCH-cDCC model was the one that generically produced the best estimating performance. However, since the specific assumptions of this model were violated in several cases, it was necessary to choose the other model.

The summary of the models selected, for each of the two situations considered (contemporary and lagged) is presented in Table 3.

**Table 3:** Bivariate cDCC-GARCH estimations

	Contemporary Analysis					Lagged Analysis				
	AE	CT	GB	PP	SW	AE	CT	GB	PP	SW
*Panel A: Model Parameters*										
$\omega$	0.0227	0.0170	0.0150	0.0202	0.0353	0.0228	0.0170	0.0151	0.0203	0.0355
	(0.0061)*	(0.0021)*	(0.0210)**	(0.0056)*	(0.0005)*	(0.0461)**	(0.0021)*	(0.0610)	(0.0056) *	(0.0005) *
$\alpha$	0.0913	0.0927	0.1170	0.0622	0.0928	0.0915	0.0928	0.1171	0.0622	0.0933
	(0.0000)*	(0.0000)*	(0.0000)*	(0.0000)*	(0.0000)*	(0.0000)*	(0.0000)*	(0.0000)*	(0.0000)*	(0.0000)*
$\beta$	0.9019	0.9019	0.8772	0.9285	0.8816	0.9017	0.9019	0.8770	0.9285	0.8811
	(0.0000)*	(0.0000)*	(0.0000)*	(0.0000)*	(0.0000)*	(0.0000)*	(0.0000)*	(0.0000)*	(0.0000)*	(0.0000)*
$\theta_{1}$	0.0076	0.0039	0.0003	0.0125	0.0058	0.0002	0.0003	0.0018	0.0053	0.0007
	(0.0023)*	(0.0035)*	(0.0114)**	(0.0359)**	(0.0042)*	(0.0455)**	(0.0482)**	(0.0046)*	(0.0153)**	(0.0082)*
$\theta_{2}$	0.9576	0.9668	0.9997	0.9447	0.9728	0.9151	0.9292	0.9005	0.9013	0.9299
	(0.0000)*	(0.0000)*	(0.0000)*	(0.0000)*	(0.0000)*	(0.0000)*	(0.0000)*	(0.0000)*	(0.0000)*	(0.0000)*
$\theta_{1} + \theta_{2}$	0.9652	0.9706	1.0000	0.9572	0.9786	0.9154	0.9296	0.9022	0.9066	0.9307

	cDCC	cDCC	DCC	cDCC	cDCC	cDCC	DCC	DCC	cDCC	cDCC
*Panel B: Diagnostics Test*										
LB (20)	22.9504	25.3922	19.7914	23.5522	27.1152	21.4561	24.9213	22.9023	21.9912	26.7711
	(0.3671)	(0.3013)	(0.4723)	(0.3512)	(0.2513)	(0.4005)	(0.3381)	(0.3700)	(0.3989)	(0.2819)
LB^2^ (20)	17.2351	18.9002	15.4123	19.1500	20.0035	16.8825	18.0024	17.8882	17.5244	19.8325
	(0.5504)	(0.4899)	(0.6535)	(0.4545)	(0.3966)	(0.6101)	(0.5119)	(0.5399)	(0.5441)	(0.4212)
ARCH (20)	15.0056	9.0015	19.1373	7.5562	4.1356	17.9946	10.7782	11.6672	13.9982	5.7726
	(0.5712)	(0.6524)	(0.3723)	(0.7089)	(0.8551)	(0.4257)	(0.6786)	(0.6345)	(0.6033)	(0.7667)

This table reports the estimation results of the bivariate models and corresponding residual diagnosis. Panel A reports the estimation results of the coefficients. The figures in parentheses (.) are p-values. Panel B reports the results of residual diagnosis. Raw residuals are used for residual diagnostic check. LB(k) and LB^2^(k) (with k = 20) are the Ljung–Box Q statistics of kth order autocorrelation for residuals and their squares, respectively. ARCH (20) denotes the Engle (1982)’s test to check for the presence of ARCH effects up-to 20 lags. *, and ** indicate significance at the 1% and 5% levels, respectively

Table 3 reports the model estimates (panel A) and related diagnostic tests (panel B) for the five environmental stock markets. Firstly, panel A of Table 3 shows that the parameters in the conditional variance equations are all statistically significant at the 1% level. The estimated value of β (GARCH effect) is close to unity (in all models the estimated values are around 0.90) and is significant at the 1% level for each model. This indicates a high degree of volatility persistence in all stock market returns.

Estimations of the bivariate models showed that the correlation of each investor sentiment/environmental segment returns is extremely persistent with correlation in the preceding period (t − 1), with significant a and b values at the 5% level, indicating that the estimates of dynamic conditional correlations are reliable. When the two values are added together the result, which in all cases is greater than 0.9, indicates that the ability to describe correlations based on previous time correlations is declining and that the correlation is not constant but changes over time.

Panel B performs the diagnostic tests that support the bivariate model’s statistical appropriateness, taking into account the insignificance of the Ljung–Box and ARCH-LM test statistics. Therefore, we can conclude that there is no misspecification in our models.

**Fig. 2:**
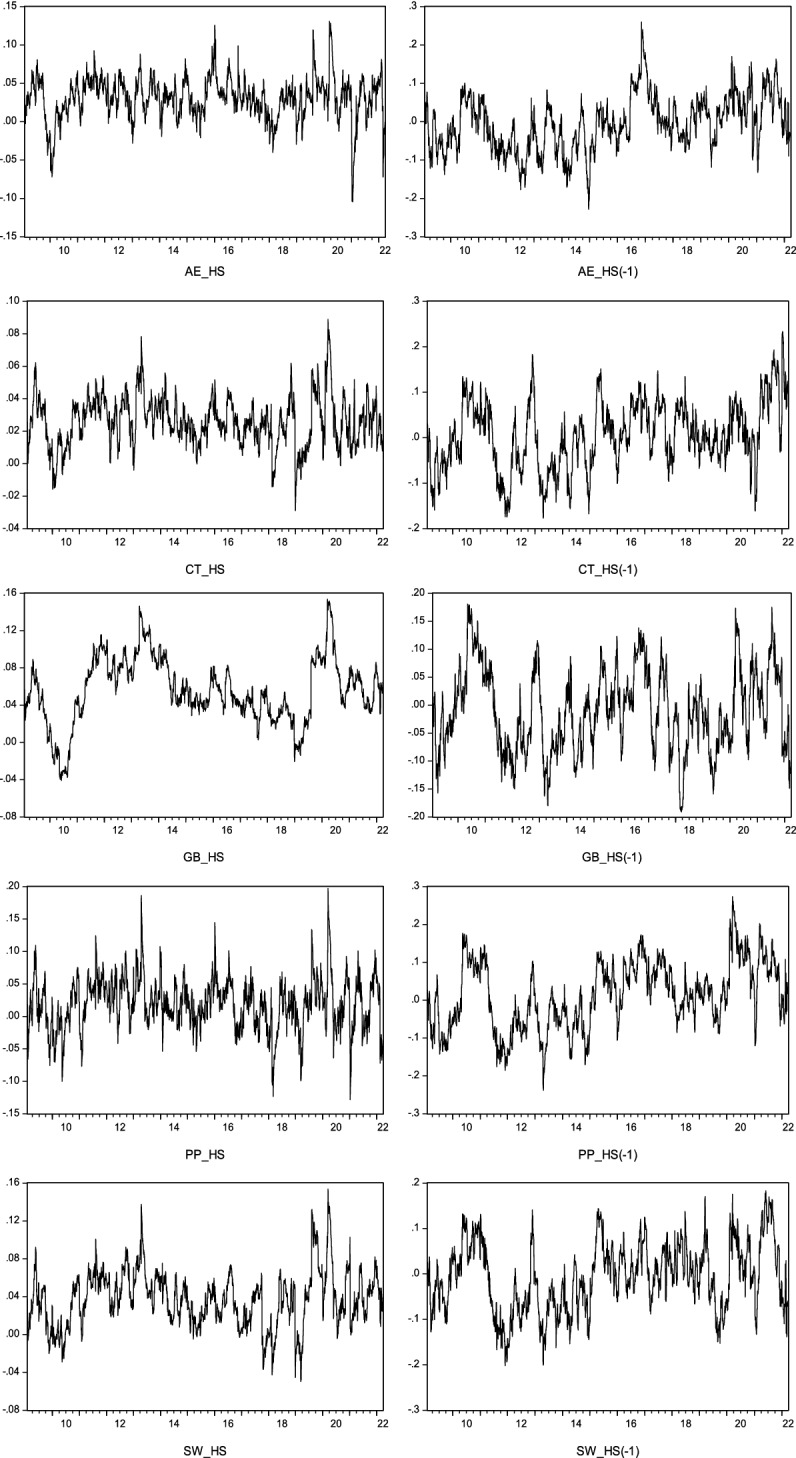
Dynamic conditional correlations

In order to analyze the correlations variable in time between the environmental segment and investor sentiment, Fig. 2 was formed for contemporary and lagged situations considering the estimates generated by the selected models, according to the specifications summarised in Table 3. Figure 2 on the left presents the contemporary dynamic correlations between the two variables. On the right, we have the dynamic correlations lagged one day in the variable of the happiness sentiment. The aim is to deepen the capacity demonstrated by this variable in providing information about future movements in the environmental segment prices. The conditional correlations showed great variability over the period studied in both of the situations considered.

Concerning the contemporary conditional correlations, positive values were recorded in the vast majority of cases, although the correlation levels found were not very high, which is consistent with the results obtained by Brown and Cliff (2004) and Oliveira et al. (2013) for conventional indices. No significant differences were identified between the five segments related to SDGs. The highest values were recorded from March 2020 onwards after the onset of the pandemic, which will have had transversal consequences for the various market segments, creating the ideal context for the general state of mind alluded to in the understanding of Baker and Wurgler (2007), and which led to sharp falls in stock market capitalizations and in investors’ expectations. In turn, the war between Russia and Ukraine, which began on 24 February, has not given rise to a significant intensification in conditional correlation levels, although the end of this event is still to be determined. Considering the results obtained, we believe that the proximity between investor sentiment and environmental segments occurs particularly in phases of major turbulence. Even so, whatever the circumstances, the correlation levels recorded between the two variables are far short of those obtained in other studies such as Fang et al. (2018) and Escobari and Jafarinejad (2019), involving traditional indices, and which are based on purely financial logic. This could mean that investment-related to SDGs is less susceptible to influence from transversal market factors.

Analysing the hypothesis of the occurrence of changes in the performance of investment segments linked to SDGs as a function of the impacts caused by investor sentiment, and considering the lag of one day in this variable, as with contemporary relations the conclusion is that the correlations present high variability overtime on a dynamic path which in any of the bivariate situations includes both positive and negative values. In addition, as in contemporary relations, no significant differences were found in the reactions of the five segments analysed concerning the happiness sentiment variable.

As for the lagged conditional correlations, and when compared to the contemporary scenario, three aspects should be highlighted. The first concerns the sharp increase in the intensity of the correlations, both positive and negative. This may mean that the state of mind on a particular day does not produce immediate effects, but rather has implications for market performance on the following day. The second aspect supports the notion that, in the absence of full information about markets, investors may make irrational decisions, letting themselves be guided by emotions, fear, and perceptions, which become important elements in the pricing of environmental assets, and which move them away from their fundamental values. This is in line with the conclusions found in other research works (Baker and Wurgler 2007; Bathia and Bredin 2012; Escobari and Jafarinejad 2019). The third aspect concerns the possible explanation for the increased intensity of negative correlations. According to Schmeling (2009), Baker et al. (2012), Bathia and Bredin (2012), and Herve, Zouaoui and Belvaux (2019), lagged investor sentiment represents a kind of risk factor of stock market segments and is the consequence of decisions taken without due grounding by some investors, possibly by so-called noise traders. These can trigger later correction to a greater or lesser degree, thereby corroborating the theses underlying the theory of behavioral finance. In this domain, there, therefore, seems to be no difference between traditional stock market sectors and sustainable sectors.

By combining the results obtained for the two scenarios considered (contemporaneous and lagged), it is possible to conclude that investor sentiment may help to explain environmental investment behaviour. This finding is fairly consistent with the results obtained in other works, such as those by Zhang et al. (2016), Shen et al. (2018), and Teti et al. (2019) using traditional indices guided purely by finance-based logic.

To check the robustness of the results for the short term, two distinct procedures were considered. First, the bivariate models of conditional heteroscedasticity were estimated considering weekly periodicity data. The results generated from these data are consistent with those presented in this paper, with no change in the dynamics established between the variables studied. Second, several specifications of the bivariate models were considered, with the time-varying correlations proving to be closely correlated with those obtained for the models presented in Table 3, confirming the robustness of the results.

To identify possible balanced and long-term relations between the variables studied, the Bounds Test was used on a data sample taken from 20 January 2009 to 29 March 2022, following the method proposed by Pesaran et al. (2001). Moreover, to contrast the robustness of the results, this research analyses whether the interconnection between the main variables studied shows a different behaviour, according to the stage of the economy, by dividing the whole sample period into two subperiods: prior to the global declaration of the Covid-19 pandemic by the World Health Organization (WHO) on 11 March 2020 and during the health crisis.

Considering the indications provided by Pesaran et al. (2001), we start by testing various formulations, or different lags, and then assess the quality of each considering the information criteria of Akaike and Schwarz. Simultaneously, the F statistic was calculated, as presented in Table 4, to test whether the joint significance of the lagged variables’ coefficients is statistically different from zero; in other words, whether the ECM is significant and, consequently, whether the hypothesis of cointegration can be accepted. Finally, the value of the F statistic was compared with the lower and upper limits of the critical values of that test. If the value of the F statistic is below the lower limit, the conclusion is the non-existence of cointegration, whereas if it is above the upper limit, the variables considered are cointegrated. When the F statistic is between those limits, the test is inconclusive.

**Table 4:** Summary of the Bounds Test results

Variables	Whole sample Period		Pre-Covid-19 Subperiod		Covid-19 Subperiod	
	ARDL Model (Optimal Lag Length)	F Statistic	ARDL Model (Optimal Lag Length)	F Statistic	ARDL Model (Optimal Lag Length)	F Statistic
AE/HS	(3;0)	0.9887	(2;0)	1.7909	(4;0)	2.1380
CT/HS	(4;0)	2.3580	(2;1)	1.5326	(1;0)	1.7740
GB/HS	(4;1)	2.1209	(4;1)	1.8694	(4;0)	2.8770
PP/HS	(2;0)	1.2646	(2;0)	1.6857	(2;0)	1.6444
SW/HS	(4;1)	1.2093	(4;3)	1.0796	(4;0)	2.2774

This table contains the Bounds Test results for each bivariate relation between the Alternative Energy Indices (AE), Clean Technology (CT), Green Building (GB), Pollution Prevention (PP), Sustainable Water (SW), and the Happiness Sentiment (HS), for the sample period from January 2009 to March 2022. The optimal lag length for the ARDL model was chosen on the basis of the information criteria proposed by Akaike and Schwarz. The critical values of the Bounds Test of 4.94 and 5.58 were taken into account for the lower limit I(0) and the upper limit I(1), respectively, according to the values presented in Table C1.iii, provided by Pesaran et al. (2001). Considering the multiplicity of the estimates produced, the authors chose not to fully include these results in the present work. If the reader is interested in accessing these estimates, they are available from the authors upon request

Table 4 presents the optimal structure of lags of the different models estimated, considering the indications provided by the information criteria of Akaike and Schwarz as well as the results of the F statistic, which reflects the joint significance of the coefficients of each pair of lagged variables in levels, of the whole sample, pre-Covid-19 and Covid-19 periods, respectively.

In all the cases considered, the statistics presented in Table 4 were below the lower bound for a 1% level of significance, which implies rejecting the hypothesis of cointegration between these variables and the sentiment indicator.

In addition, the results obtained for the long-term lead to the conclusion that environmental segments and investor sentiment follow autonomous paths, and do not generate balanced relations between the two variables, in none of the sample sub-periods, unlike what has been reported in other research, such as Schmeling (2009), although these were obtained from traditional stock market indices. The non-existence of a common stochastic tendency between the two types of variables suggests that the relation between these environmental segments, which incorporate companies linked to the SDGs, and investor sentiment was not marked by stability over the period analysed. Taking into account the results obtained, we, therefore, conclude that sentiment waves, in the sense of Schmeling (2009) and Finter et al. (2011), which cause asset prices to deviate from their fundamental value, have a mainly short-term effect and that environmental asset prices tend to approach their fundamental value in the long run, reducing the intensity of the relationship between them and investor sentiment.

These results may also imply certain differences compared to traditional indices. Although volatility and, sometimes, turbulence are elements that characterize the behavior of stock markets in general, the fact that environmental investment is based on values that are not restricted to the financial issue may cause the weight of the rational investment process, guided by sustainable development goals, to override the emotional process which is usually present in investors' decisions.

To test the robustness of the results, Johansen's (1998) proposal was adopted, based on the trace and maximum eigenvalue tests. However, similar to the results obtained using the method proposed by Pesaran et al. (2001), no cointegrating vectors were identified, thereby reinforcing the robustness of the estimates.

## Final considerations

This research studies the relation and the stability of the relationship generated between happiness sentiment and five environmental stock market segments, which incorporate the SDGs set out by the United Nations. To achieve the aims of the study, the bivariate relations provided by the two variables were analysed for approximately twelve years using a diversified methodological proposal involving the Bounds Test and models of conditional heteroscedasticity.

The research option first allowed us to reflect on investor sentiment, which is a relatively new and thus far underexplored factor and which breaks with traditional theory. Second, it enabled us to study in greater depth the behaviour of some of the most recent indices of a sustainable nature whose characteristics differentiate them from traditional indices. We thus merged two elements not yet studied in the literature, particularly the inclusion of a new proxy for investor sentiment and its relation with environmental investment.

To analyse the short-term dynamics between the two variables, various models of dynamic conditional correlation were estimated. This provided empirical evidence that the conditional correlations present high variability, with phases of greater turbulence, as experienced during the outbreak of the pandemic. This indicates closer paths between investor sentiment and environmental investment.

Considering the impact on environmental segments caused by the lagged sentiment variable, it was possible to conclude that, similar to the conclusions reached in other research obtained from traditional indices such as Bathia and Bredin (2012), Schmeling (2009), Baker et al. (2012) and Herve, Zouaoui and Belvaux (2019), the impact caused by the sentiment variable on market performance was negative. This leads us to suppose that investor sentiment can be a kind of risk factor for environmental stock market segments which does not allow them to be distinguished from traditional indices.

In all the cases, the Bounds Test led to the conclusion that the bivariate relations between the environmental segments and investor sentiment did not show signs of cointegration, which is one reason for believing that these variables follow autonomous paths. This implies testing for a differentiated pattern of behaviour between the two variables, each of which has a specific and individualized generating mechanism. The absence of identical movements may mean that the behaviour of these variables is not guided by common factors or that it is marked by a common stochastic tendency.

In line with the results obtained, it is plausible to consider that the relationship between sentiment and environmental investment is not marked by inseparability but rather by episodic situations that are delimited in time and which are formed above all during times of turbulence.

The results obtained here provide valuable information for academics, investors, and regulators, giving new perspectives on the current debate concerning the question of the rationality or irrationality of sentiment, the production of long and short-term effects, and the generation of permanent or temporary effects, or even causing homogeneous or heterogeneous effects on investment segments related to the SDGs. Similarly, it helps to understand whether sentiment is relevant in modelling asset prices, investment analysis, and in-market efficiency policy. The results of this study support the importance of investor sentiment in that this can influence stock market behaviour, economically and statistically. Concerning future academic research, other work can be carried out, particularly in terms of further exploring the assumptions of behavioral theory and identifying other proxies for the sentiment that are shown to be better adjusted to true market sentiment. In future research, we also intend to go deeper into the connection between investor sentiment and investment segments linked to the SDGs, first by using other methodological approaches; namely, the structural vector autoregressive model and the concept of spectral causality. This will allow a more detailed analysis of the dynamics formed between the two investment segments. Secondly, we aim to extend the sample period to understand more comprehensively the impact of the pandemic and the Russia-Ukraine war on the association between the variables studied. It will also be interesting to find out whether this crisis contributed to an awareness of sustainable investment.

## Data Availability

My manuscript has no associate data.

## Declarations

### Human and animal Participants

Research involving Human Participants and/or Animals NA.
